# Preventive effect of metronidazole vaginal tablets on vaginal bacteria-related postoperative complications with total laparoscopic hysterectomy

**DOI:** 10.1186/s13256-023-03789-1

**Published:** 2023-02-14

**Authors:** Asuka Okamura, Wataru Isono, Akira Tsuchiya, Michiko Honda, Ako Saito, Hiroko Tsuchiya, Reiko Matsuyama, Akihisa Fujimoto, Osamu Nishii

**Affiliations:** Department of Obstetrics and Gynaecology, University Hospital Mizonokuchi, Teikyo University School of Medicine, 5-1-1, Futago, Takatsu-Ku, Kawasaki, Kanagawa 213-8507 Japan

**Keywords:** Total laparoscopic hysterectomy, Multivariate analysis, Retrospective study, Vaginal bacteria-related postoperative complications, Metronidazole vaginal tablet

## Abstract

**Background:**

The use of total laparoscopic hysterectomy is increasing. However, as with conventional abdominal hysterectomy, vaginal bacteria-related postoperative complications need to be managed in total laparoscopic hysterectomy. Therefore, we started to combine metronidazole vaginal tablets with intravenous administration of cephem antibiotics immediately before starting surgery to reduce complications. To evaluate the effect of this combination, and to determine the risk factors for these complications, we retrospectively collected medical records from our hospital and performed a multivariate analysis.

**Methods:**

We reviewed the medical records of 520 patients who underwent total laparoscopic hysterectomy from 1 January 2015 to 31 December 2021. Among these cases, we identified 16 cases as having vaginal bacteria-related postoperative complications, defined as needing more than one additional day for treatment of postoperative complications, namely postoperative infection (10 cases) and vaginal dehiscence (6 cases). First, we evaluate the effect of metronidazole vaginal tablets by dividing the patients into two groups according to whether metronidazole vaginal tablets were used, and comparing the vaginal bacteria-related postoperative complication rates and other indices. Second, we performed a multivariate logistic regression analysis to assess the influence of each of 17 representative factors, including patient characteristics and symptoms, uterus and leiomyoma sizes, concomitant procedures such as laparoscopic cystectomy and pelvic lymphadenectomy, and others.

**Results:**

In the multivariate analysis of the 520 cases, we confirmed that the use of metronidazole vaginal tablets could reduce the vaginal bacteria-related postoperative complications rate by more than half (odds ratio, 0.36). In addition to metronidazole vaginal tablets use, concomitant laparoscopic cystectomy and blood transfusion were associated with significant increases in the vaginal bacteria-related postoperative complication rate.

**Conclusions:**

The effect of the addition of metronidazole vaginal tablets to pre- and postsurgical treatment on the reduction of vaginal bacteria-related postoperative complications was confirmed. This easy, safe, and low-cost method may improve the management of total laparoscopic hysterectomy.

## Background

Since total laparoscopic hysterectomy (TLH) is associated with less pain and a quicker recovery than conventional abdominal hysterectomy (AH), the number of indications for TLH has increased, and TLH has become more widespread [1–3]. TLH is also considered to be advantageous in terms of fewer wound infections, and fewer febrile episodes or unspecified infections [4], but there is no evidence of a difference in the occurrence of vaginal cuff dehiscence [4, 5]. For this reason, also when performing TLH, further improvements may be needed to reduce postoperative complications related to the ascending spread of vaginal bacteria [6]. In some reports, antiseptic vaginal preparation for hysterectomies with disinfectant agents, such as povidone–iodine, is recommended [7, 8], and other researchers have reported that surgical site infections could be reduced by adding metronidazole, with intravenous administration of cephem antibiotics, before starting the hysterectomy [9]. On the basis of these reports, we hypothesize that both systemic and local administration of antibiotics are effective, and we adopted the combination of these drugs [10]. In our hospital, beginning in 2017, in addition to cephem antibiotics administered immediately before starting TLH, we added metronidazole vaginal tablets (MVTs) before and after the operation. Since this is an easy, non-invasive, and low-cost approach, this combination could widely provide safer management of TLH. Therefore, in this report, we aimed to confirm the effect of this procedure in a retrospective analysis of patients who underwent TLH.

## Methods

### Data collection

This study was reviewed and approved by the Human Ethical Committee of the University of Teikyo Hospital (Trial registration number 20-094). The deidentified medical records of 520 female patients who underwent TLH from 1 June 2015 to 31 December 2021 were reviewed retrospectively. In these cases, bilateral salpingectomy (BS) or bilateral salpingo-oophorectomy (BSO) was performed during TLH. This study also included 26 cases with concomitant pelvic lymphadenectomy (PLA), and 29 cases with concomitant laparoscopic cystectomy (LC), including 4 bilateral LCs and 25 unilateral LCs. All operations were performed under the direct supervision of at least one of two laparoscopic surgery experts (A.F. and O.N.). Most indications for TLH were leiomyoma (403 cases) or adenomyoma (60 cases). However, since we focused mainly on vaginal disinfection, we did not exclude other indications, including early-stage endometrial carcinoma (31 cases), cervical intraepithelial neoplasia (11 cases), atypical endometrial hyperplasia (8 cases), uterine cervical tumor (4 cases), vaginal atresia with menstrual molimen (1 case), cesarean scar pregnancy (1 case), or cesarean scar syndrome (1 case). We extracted data on patient characteristics, such as age, delivery history, presenting symptoms, and physical examination findings, from medical records, since these factors were considered to be related to TLH outcomes. Surgical history and intraoperative findings were also included, since these factors might have an influence on the difficulty of performing a TLH, especially the process of suturing the vaginal wall. To evaluate the effect of MVT on TLH, we extracted data on the vaginal bacteria-related postoperative complications (VBRPC) of patients, including postoperative infection (ten cases) and vaginal dehiscence (six cases), for whom the diagnosis was made within 30 days after the operation [9], and an additional outpatient or hospitalization period of at least 1 day was needed. As a limitation of this study, we performed microbiological culture tests of vaginal discharge, including *Enterococcus faecalis*, *Prevotella loescheii*, and Group B *Streptococcus*, for only 6 out of the 16 patients; therefore, postoperative infection was instead defined as C-reactive protein positivity (> 0.30 mg/dL) (7.34 ± 4.89, 1.06–15.73 mg/dL, *n* 10) and antibiotic administration. At that time, we excluded three patients, in which bleeding, hemorrhage, or an abscess was present in the trocar sites.

### Analysis methods

First, to evaluate the effect of MVT (Flagyl 250 mg, Fuji Pharma Co., Ltd., Tokyo, Japan), we divided the patients into two groups according to the presence or absence of the use of this tablet (425 versus 95 cases). In our hospital, we administered three vaginal tablets to the former patients, timed at 1 day before TLH, immediately after completion of the operation, and 3 days after the operation. In these two groups, we compared the 17 indices presented in Table 1 by using Student’s *t*-test and Pearson’s chi-square test. Second, to confirm the effect of vaginal tablets on VBRPC, we tried to identify risk factors for VBRPC. To control confounding factors, we divided the patients into two groups according to the presence or absence of each factor, and performed multivariate logistic regression analysis. In this analysis, we assessed the influence of the following 17 factors: (1) advanced age, defined as an age ≥ 50 years; (2) high body mass index (BMI), defined as a BMI ≥ 25 (kg/m^2^); (3) nulliparity, defined as no previous delivery; (4) gynecological operation history; (5) menstrual disorder, defined as patients with menstruation-related symptoms such as dysmenorrhea, menostaxis, menorrhagia, anemia or abnormal vaginal bleeding; (6) abdominal distension, defined as patients with abdominal symptoms such as abdominal pressure, pelvic pain, dysuria, or dyschezia; (7): GnRH analog, defined as treatment with gonadotropin-releasing hormone analog (GnRHa) before TLH; (8) concomitant LC; (9) concomitant PLA; (10) long-time operation, defined as an operation lasting over 240 minutes according to the average and standard deviation of 520 cases; (11) massive blood loss, defined as loss of over 500 mL of blood; (12) heavy uterus, defined as a resected uterine weight ≥ 500 g; (13) abdominal adhesion, defined as abdominal adhesion detected by laparoscopic inspection immediately after the start of surgery; (14) blood transfusion, defined as the need for blood transfusion, including both autologous and allogeneic blood transfusion, during or after the operation; (15) large leiomyoma, defined as a dominant leiomyoma ≥ 8 cm by magnetic resonance imaging (MRI); (16) large uterus, defined as an average uterine length ≥ 10 cm by transvaginal ultrasound (TVUS) before surgery; and (17) MVT use, defined as treatment with vaginal tablets. The criteria for “massive blood loss,” “large leiomyoma,” and “heavy uterus” were determined on the basis of past reports [11–13]. Statistical analyses were performed using Microsoft Excel (Microsoft Corporation, Redmond, WA) and JMP version 12 for Windows (SAS Institute, Inc., Tokyo, Japan) to determine the correlations between patient characteristics and VBRPC. The odds ratios (ORs) and 95% confidence intervals (CIs) were estimated to determine the strength of the correlations. *p* < 0.05 was considered statistically significant.

**Table 1:** Effect of metronidazole vaginal tablet

Index	No use	MVT use	*p* value
Age (years old)	47.9 ± 7.7 (35–81)	47.3 ± 5.4 (54–73)	NS
BMI (kg/m^2^)	23.0 ± 3.6 (15.9–37.8)	22.9 ± 3.9 (15.1–46.5)	NS
Nulliparity	*n* 28/95	*n* 168/425	NS
Gynecological operation history	*n* 20/95	*n* 88/425	NS
Menstrual disorder	*n* 76/95	*n* 330/425	NS
Abdominal distension	*n* 13/95	*n* 119/425	< 0.01
GnRH analog	*n* 47/95	*n* 306/425	< 0.01
Concomitant LC	*n* 6/95	*n* 23/425	NS
Concomitant PLA	*n* 6/95	*n* 20/425	NS
Operation time (minutes)	198.2 ± 53.1 (94–361)	206.0 ± 56.2 (99–504)	NS
Blood loss (mL)	135.7 ± 185.6 (0–1189)	121.6 ± 200.9 (0–2003)	NS
Uterine weight (g)	257.5 ± 155.8 (50–925.2)	324.4 ± 234.7 (28–1454)	< 0.05
Abdominal adhesion	*n* 35/95	*n* 182/425	NS
Blood transfusion	*n* 5/95	*n* 19/425	NS
Leiomyoma size (cm)	5.7 ± 2.4 (1.7–12.5)	6.5 ± 3.1 (1.1–20.0)	< 0.05
Uterine size (cm)	7.0 ± 1.7 (2.9–11.9)	7.4 ± 2.0 (3.0–15.5)	NS
VBRPC	*n* 6/95	*n* 10/425	< 0.05

After dividing 522 patients into two groups according to whether MVTs were used, we compared 17 representative indices. In this analysis, five indices, namely abdominal adhesion, GnRH analog, uterine weight, leiomyoma size, and VBRPC, showed significant differences

*MVT* metronidazole vaginal tablet, *NS* no significance, *BMI* body mass index, *GnRH* gonadotropin-releasing hormone analog, *LC* laparoscopic ovarian cystectomy, *PLA* pelvic lymphadenectomy, *VBRPC* vaginal bacteria-related postoperative complications

## Results

### Patient characteristics

The average age, BMI, parity, and hospitalization duration of the included patients were 47.4 ± 5.9 (34–81) years, 23.0 ± 3.8 (15.1–46.5) kg/m^2^, 1.1 ± 1.0 (0–4), and 6.1 ± 2.0 (4–40) days, respectively. In total, 108 patients had a gynecological operation history, including patients with only cesarean section (36 cases), and 353 patients treated with GnRHa before surgery. The overall average operation time of the 520 patients was 204.6 ± 55.6 (94–504) minutes, the average blood loss volume was 124.1 ± 198.1 (0–2003) mL, and the average weight of the resected uterus was 312.3 ± 223.9 (28–1454) g. Of the 520 patients, 60 had no complaints, but the other 460 patients had various symptoms, such as menstrual disorder and abdominal distension; one patient reported multiple symptoms. By MRI, the average size of the dominant leiomyoma in 447 patients was 6.3 ± 3.0 (1.1–20.0) cm, and submucous leiomyoma was found in 108 cases. The average hemoglobin concentrations before and after the operation were 13.0 ± 1.2 (6.6–16.7) and 11.5 ± 1.3 (5.1–15.2) g/dL, respectively. Autologous blood donation was performed in 271 patients, and blood transfusion was performed in 24 patients, including 21 patients who required only autologous blood transfusion, 1 patient who required allogeneic blood transfusion, and 2 patients who required both.

### Effect of metronidazole vaginal tablet

To evaluate the effect of MVT, we compared two groups of patients classified according to whether they received MVT. As expected, the rate of VBRPC was significantly lower in patients who received MVT (Table 1). However, significant differences were also detected in the other four indices, namely abdominal distension, GnRH analog, uterine weight, and leiomyoma size. Therefore, to identify significant factors affecting the VBRPC rate without the influence of confounding factors, multivariate analysis of 17 representative factors was performed (Table 2). In this analysis, we found that MVT use was significantly negatively associated with the rates of these complications in this analysis (OR 0.36, 0.13–1.00, *p* < 0.05). Apart from this result, concomitant LC (OR 4.24, 1.14–15.81, *p* < 0.05) and blood transfusion (OR 5.31, 1.40–20.05, *p* < 0.01) showed significant differences. Due to the above-mentioned results, we confirmed the effectiveness of MVT.

**Table 2:** Identification of influential factors for vaginal bacteria-related postoperative complication

Factors	Number	OR (95% CI)	*p* value
Advanced age	125	0.72 (0.20–2.58)	NS
High BMI	129	0.42 (0.10–1.89)	NS
Nulliparity	196	1.30 (0.47–3.54)	NS
Gynecological operation history	108	0.88 (0.25–3.13)	NS
Menstrual disorder	406	0.45 (0.16–1.28)	NS
Abdominal distension	132	1.80 (0.64–5.05)	NS
GnRH analog	353	1.43 (0.46–4.51)	NS
Concomitant LC	29	4.24 (1.14–15.81)	*p* < 0.05
Concomitant PLA	26	1.28 (0.16–10.06)	NS
Long-time operation	65	1.65 (0.46–5.94)	NS
Massive blood loss	22	1.53 (0.19–12.16)	NS
Heavy uterus	82	1.24 (0.35–4.46)	NS
Abdominal adhesion	217	0.63 (0.21–1.83)	NS
Blood transfusion	24	5.31 (1.40–20.05)	*p* < 0.01
Large leiomyoma	78	1.02 (0.32–3.22)	NS
Large uterus	51	1.33 (0.29–6.01)	NS
MVT use	425	0.36 (0.13–1.00)	*p* < 0.05

A multivariate analysis of 522 patients was performed to examine the influence of 17 representative factors that were collected from medical records. The number of patients with each factor, the ORs and 95% CIs for the occurrence of these complications, and the *p* values are presented in this table. “MVT use,” “concomitant LC,” and “blood transfusion” were identified as significant factors for the occurrence of these complications

*OR* odds ratio, *CI* confidence interval, *NS* no significance, *BMI* body mass index, *GnRH* gonadotropin-releasing hormone analog, *LC* laparoscopic ovarian cystectomy, *PLA* pelvic lymphadenectomy, *MVT* metronidazole vaginal tablet

## Discussion

Since surgeons must cope with both skin and vaginal bacteria when performing hysterectomy [6, 9, 10], in our hospital, we started to use MVTs in addition to conventional intravenous administration of cephem antibiotics immediately before starting the operation. We created our original protocol according to previous research studies that evaluated the prophylactic effect of the additional use of intravenous metronidazole on postoperative infection, in which a single dose did not have a significant effect [11], but multiple doses had a significant effect [14]. This combination was hypothesized to reduce postoperative complications, related to the ascending spread of vaginal bacteria for TLH. Then, to evaluate its effectiveness, we extracted data on 16 cases with postoperative infection (10 cases) or vaginal cuff dehiscence (6 cases) as the index for VBRPC; these patients needed additional treatment time of at least one day (5.2 ± 8.0, 1–34 days, *n* 16). However, we observed a retrospective study-specific limitation, and could not strictly detect a causal relationship with infection by performing a microbiological culture test of vaginal discharge. The rate of VBRPC reached approximately 3% (*n* 16/520) and was approximately equal to those of previous reports [9, 15], but we also detected one severe case, in which the patient needed laparoscopic drainage to treat a severe abdominal abscess. In this study, to compare the rates of VBRPC of the two groups of patients classified according to the presence or absence of MVT use, we performed multivariate analysis of 17 factors, including MVT use. To prevent the arbitrary selection of factors, we included as many factors as we could collect from electronic medical records, which possibly affected the outcomes of the operations, including the patients’ physical and clinical characteristics; although, there was the possibility of overselection or inappropriate selection of factors. In this analysis, MVT use had a significant effect, reducing the VBRPC rate by more than half (OR 0.36, 0.13–1.00). This result supports the notion of recommending this combination for infection prevention management in TLH, since MVT is safe, low-cost, and non-invasive, and antibiotic resistance against metronidazole is thought to be relatively rare [9, 16]. Therefore, we suggest further prospective study on proactive MVT use. We also detected a significant increase in the rates of “concomitant LC” or “blood transfusion” in this multivariate analysis. These results are consistent with previous reports, as massive blood loss and ovarian cyst content are risk factors for an inflammatory response [17, 18]. On the other hand, “concomitant PLA” did not show a significant difference. Although we could not determine the influence of the indications for TLH, the presence of a malignant tumor hardly influences the rate of VBRPC.

## Conclusions

This study assessed the effect of the additional administration of MVTs on reducing vaginal bacteria-related postoperative complications. This easy, safe, and low-cost method has the possibility of improving the management of preventing TLH-related infections.

## Data Availability

The authors agree to make all data in this study freely available.

## Abbreviations

TLH: Total laparoscopic hysterectomy
AH: Abdominal hysterectomy
MVT: Metronidazole vaginal tablet
BS: Bilateral salpingectomy
BSO: Bilateral salpingo-oophorectomy
PLA: Pelvic lymphadenectomy
LC: Laparoscopic cysterectomy
VBRPC: Vaginal bacteria-related postoperative complications
BMI: Body mass index
GnRHa: Gonadotropin-releasing hormone analog
MRI: Magnetic resonance imaging
TVUS: Transvaginal ultrasound
OR: Odds ratio
CI: Confidence interval
